# Water vapor absorption allows for volume expansion during molting in *Armadillidiumvulgare* and *Porcelliodilatatus* (Crustacea, Isopoda, Oniscidea)

**DOI:** 10.3897/zookeys.801.23344

**Published:** 2018-12-03

**Authors:** John-David Nako, Nicole S. Lee, Jonathan C. Wright

**Affiliations:** 1 Department of Biology, Pomona College 175 West 6th Street Claremont, CA 91711, USA Pomona College Claremont United States of America; 2 UCLA School of Dentistry 10833 Le Conte Ave, Los Angeles, CA 90095, USA UCLA School of Dentistry Los Angeles United States of America

**Keywords:** Isopoda, Oniscidea, water vapor absorption, molting, ecdysis

## Abstract

Arthropods require periodic molting in order to grow which presents a number of challenges to terrestrial taxa. Following ecdysis, the pliant new cuticle is susceptible to buckling under gravity and requires elevated hydrostatic pressure for support. Terrestrial species also require a mechanism of volume expansion and stretching of the integument prior to sclerotization, a need that is readily met in aquatic arthropods by drinking. Options for land arthropods include drinking of dew, swallowing of air, or using muscular contractions to inflate air sacs in tracheate taxa. In this study we tested the hypothesis that crinochete terrestrial isopods (Isopoda: Oniscidea: Crinocheta) exploit their capacity for active water vapor absorption (WVA) to increase volume during molting. Two crinochete species, *Armadillidiumvulgare* and *Porcelliodilatatus*, were studied and compared with the non-absorbing species *Ligidiumlapetum* (Oniscidea: Ligiamorpha). Pre-molting animals were identified by sternal CaCO_3_ deposits and exposed to 100% or 97% relative humidity (RH). Mass-changes were monitored by daily weighing and the timing of the posterior and anterior ecdyses was used to categorize time (days premolt and days post-molt) over the molt cycle. In each treatment RH, *A.vulgare* and *P.dilatatus* showed a progressive mass increase from 5 days premolt until the posterior or anterior ecdysis, followed abruptly by period of mass-loss lasting 3–4 days post-molt. The fact that the initial mass-gain is seen in 97 % RH, a humidity below the water activity of the hemolymph, confirms the role of WVA. Similarly, since the post-molt mass-loss is seen in 100 % RH, this must be due to active expulsion of water, possibly via maxillary urine. Concurrent changes in hemolymph osmolality were monitored in a separate batch of *A.vulgare* and show sustained osmolality during premolt and an abrupt decrease between the anterior and posterior ecdysis. These patterns indicate a mobilization of sequestered electrolytes during premolt, and a loss of electrolytes during the post-molt mass-loss, amounting to approximately 8.6 % of total hemolymph solutes. WVA, in conjunction with pulses of elevated hemolymph pressure, provides an efficient mechanism of pre-molt volume expansion prior to and during the biphasic molt in these species. Premolt *Ligidiumlapetum* exposed to same treatments failed to molt successfully and no premolt animals survived to day 3 (72 h) even in 100 % RH. The apparent dependence of this species on liquid water for successful molting could explain its obligatory association with riparian fringe habitats.

## Introduction

The cuticle of arthropods is an organ of extraordinary adaptive versatility, allowing for articulation and movement via complex joints, sensory transduction using a remarkable variety of permeable or deformable sensilla, variable morphology and coloration from impregnated pigments or refractory laminae (physical coloration), and extreme resistance to water loss from intrinsic or superficial lipids in many terrestrial taxa (Locke 1965, 1974, Chapman 2012). The adaptive plasticity of the cuticle has doubtless contributed to the spectacular adaptive radiations of the insects, arachnids and myriapods in terrestrial habitats. However, the requirement that the cuticle be periodically molted and renewed to allow for growth presents a number of challenges to land colonization.

Molting of the arthropod exoskeleton is preceded by apolysis – the separation of the old cuticle from the underlying epidermis – and the secretion of inactive molting fluid via dermal glands prior to the secretion of the cuticulin layer in the presumptive new cuticle (see Locke 1974, Chapman 2012). Subsequent activation of the molting fluid results in degradation and resorption of the old endocuticle (Samuels and Reynolds 1993). At this stage, the new cuticle is soft and flexible to allow for expansion and growth. An increase in hemolymph hydrostatic pressure precedes ecdysis during which the old cuticle splits along well-defined ecdysial lines and is sloughed. Volume expansion of the newly molted animal typically continues for a few hours to a few days until distension of the new cuticle is inhibited by progressive quinone tanning of the endocuticle proteins and/or mineralization (Fraenkel and Rudall 1947, Vincent and Hillerton 1979). During the intervening period, the soft, pliant cuticle renders the animal vulnerable, limits mobility, and requires that terrestrial species generate elevated hydrostatic pressure for structural support (DeFur et al. 1985, Chapman 2012). Pliant intersegmental cuticle may continue to expand between molts, allowing for a sustained size increase, but mineralized or sclerotized cuticle does not expand. This was well illustrated by Clarke (1957) who showed that intermolt tibia length remains constant in *Locusta*, then increases abruptly at ecdysis, despite a steady increase in whole-animal mass. Volume increase and growth of the whole animal thus depends, in part, on the expansion of the cuticle during molting.

Details of the hemolymph volume and localized pressure increases accompanying ecdysis have been studied in several insects and aquatic crustaceans. Molting flies and locusts are thought to swallow air to bring about volume expansion (Ewer 1954, Clarke 1957, Cottrell 1962a,b, Cottrell 1964, Lee 1961). Following pupal eclosion, the blowflies *Calliphora* and *Sarcophaga* swallow air which distends the gut, producing a steady increase in hemolymph hydrostatic pressure. Simultaneous contractions of specialized ptilinal and abdominal muscles generate pressure pulses involved in wing expansion (Cottrell 1962a,b). These muscles degenerate a few days following ecdysis. Specialized muscles functioning to create localized pressure increases during molting have been identified in several insects, some of them associated with specific instars (Ewer 1954, Miyan 1989). The hemolymph volume at emergence of adult blowflies is comparable to that of the larvae, and hydrostatic pressure provides structural support to the pharate adult. Hemolymph volume falls dramatically (by about 80 %) over the succeeding 30 hours, as the cuticle is stiffened by tanning. This is accompanied by compensatory intake of air into the thoracic air sacs and some fluid release from the anus (Wigglesworth 1963).

Aquatic crustaceans drink water to increase hemolymph volume prior to and following ecdysis (Dall and Smith 1978, Hartnoll 1982, 1988, DeFur et al. 1985, 1988). In marine decapods, drinking of seawater begins about 1 hour prior to ecdysis and continues for 4–7 hours after, depending on species (Travis 1954, Robertson 1960, Mykles 1980). In juvenile *Homarusamericanus*, ingested seawater is absorbed across the midgut and brings about an increase in hemolymph volume of 46 % prior to ecysis, and 167 % 2.5 h following ecdysis (Mykles 1980). During the succeeding 2 weeks, hemolymph volume falls steadily while intracellular water and dry mass increase. The net mass-gain at completion of the molt is about 30 %. A similar process is seen in the euryhaline blue crab *Callinectessapidus* (Neufeld and Cameron 1994) and in the amphipod *Gammarusduebeni* (Lockwood and Inman 1973), and the fraction of branchial water uptake (ca. 30 %) is similar in *Callinectes* acclimated to 2 ‰ and 28 ‰; decreased osmotic permeability restricts water uptake in the lower salinity. As with *Homarus*, imbibed water in *Callinectes* (Neufeld and Cameron 1994), and in *Carcinusmaenas* (Robertson 1960), moves into the hemolymph across the midgut cecae.

Oral uptake of water similarly provides the main mechanism of volume expansion during molting in freshwater and athassohaline crustaceans but imposes an osmotic challenge. Hemolymph osmolality and specific ion concentrations decline post-molt relative to intermolt in the athassohaline Chinese crab *Eriocheirsinesis* (DeLeersnyder 1967), whiteleg shrimp *Litopenaeusvannamei* (Cheng et al. 2002), and the freshwater crayfish *Cheraxdestructor* (Zare and Greenaway 1998). Compensatory up-regulation of branchial ion transport during molting occurs in *Callinectes* acclimated to low salinities (Towle and Mangum 1985), and in the freshwater crayfish *Cheraxdestructor* (Zare and Greenaway 1998), and involves increased activity of both the basolateral Na^+^/K^+^-ATPase and increased apical membrane potentiation via up-regulation of the H^+^ V-ATPases.

Mechanisms of volume expansion during molting in terrestrial arthropods other than insects remain under-investigated. Some groups may drink like aquatic crustaceans, but a dependable liquid water source is often not available. Semi-arid grasslands, mountain rain shadows and continental deserts are just three examples of habitats that frequently remain above dew-point temperatures for weeks or months at a time (Cloudsley Thompson 1991, Edney 1977, Hadley 1994). Furthermore, feeding is generally precluded between apolysis and a variable period following ecdysis by the renewal of the cuticular structures overlying the ectodermally derived foregut and hindgut epithelia (Ellis 1951, Chapman 2012). Food intake is therefore probably not a viable means of volume expansion, except perhaps for some fluid-feeders. The present study was undertaken to examine the possibility that active water vapor absorption serves as a mechanism of volume expansion in the terrestrial isopods (Suborder Oniscidea).

Together with a few species of talitrid amphipods (Duncan 1994, Hou and Li 2003), the oniscidean isopods are the only crustaceans that can live truly independently of liquid water (Little 1983, 1991). Both of these groups brood the eggs in a fluid-filled marsupium (Hoese 1984, Hoese and Janssen 1989, Pandian 2016). Like other isopods, the Oniscidea have a biphasic molt in which the posterior half of the cuticle is molted first, followed after 1–2 days by the anterior half (George and Sheard 1954); the intervening period constitutes the intramolt. Biphasic molting confers advantages including improved mobility during the molt period and a limit on newly exposed permeable surface area (Price and Holdich 1980a,b). Formation of the marsupium in gravid females involves a specialized parturial molt (George and Sheard 1954, Moreau and Rigaud 2002). Prior to ecdysis, oniscideans reabsorb calcium carbonate from the old cuticle and sequester it as amorphous calcium carbonate spherules in the subcuticular space of the first four pereonal sternites (Steel 1993, Ziegler 1996, Ziegler and Miller 1997). Sequestered calcium is re-mobilized following ecdysis, allowing for rapid mineralization of the new endocuticle. The calcium deposits are conspicuous and allow for identification of pre-molt animals as well as differentiation of parturial molt females (Moreau and Rigaud 2002).

Changes in hemolymph pressure, volume and ion composition during molting in oniscideans have been studied by a few workers. Price and Holdich (1980b) measured whole-animal mass and hemolymph osmolality during molting in *Oniscusasellus*, and did not identify significant changes between 7 days prior and 7 days following ecdysis. They concluded that localized increases in blood pressure brought about by contractions of somatic musculature, rather than changes in hemolymph volume, generate the requisite pressure for the posterior and anterior ecdyses. Heeley (1941) described rhythmic inter-segmental contractions in the anterior segments preceding the posterior edysis, and subsequent contractions in the posterior segments preceding the anterior molt. Later measurements of hemolymph blood pressure in *Porcelliospinicornis* and *Armadillidiumvulgare* by Alikhan (1983, 1984) showed that regular pulses of elevated pressure appear a few minutes in advance of the posterior molt, persist intermittently during the intramolt period, and resume for 2–7 h after the anterior ecdysis. However, changes in blood pressure without accompanying increases in volume cannot explain net growth. Measurements of hemolymph electrolytes during the molt cycle in *Porcellioscaber* (Ziegler and Scholz 1997) showed that concentrations of K^+^, Na^+^, Mg^2+^ and Cl- all decrease significantly following the posterior molt, consistent with the uptake of water. Furthermore, direct measurements of hemolymph volume in the supra-littoral oniscidean *Ligiapallassii* show a sharp increase in volume following the posterior ecdysis, apparently due to direct uptake of seawater (Ziegler and Pennings 2000).

Active water vapor absorption (WVA) provides a potential mechanism for volume expansion in a few families of terrestrial arthropods (Machin 1983, O’Donnell and Machin 1988). WVA is defined as any energy-dependent process allowing an animal to absorb water vapor from a vapor pressure below the equilibrium vapor pressure of the animal’s body fluids – or from a relative humidity (RH) below about 99 % for practical purposes. The capacity for WVA has evolved independently in several arthropod lineages, including lepismatids, tenebrionid beetles, fleas, corydiid cockroaches, booklice and biting lice, oniscidean isopods, mites and penicillate millipedes (see Machin 1983, Knülle 1984, O’Donnell and Machin 1988, Gaede 1991, Wright and Westh 2006). Whether WVA plays a role during the molt cycle in any of these groups is unknown, although some species lose the capacity for WVA shortly before ecdysis (Edney 1966, Noble-Nesbitt 1978, Coutchié and Crowe 1979). The sites and mechanisms of vapor absorption vary widely but all involve the depression of water vapor pressure (and hence water activity and free energy) at a specialized absorption site (O’Donnell and Machin 1988). In the oniscidean isopods, uptake involves the secretion of strongly hyperosmotic fluid into the pleoventral (branchial) cavity which drives colligative condensation of water above an absorption threshold of 86–93 % RH depending on species (Wright and O’Donnell 1992, Wright and Machin 1993).

In this study, we set out to test whether WVA serves in volume expansion during molting in two species of oniscidean isopods, *Armadillidiumvulgare* (Latreille, 1804) (Armadillidiidae) and *Porcelliodilatatus* Brandt, 1833 (Porcellionidae). Both belong to the section Crinocheta, a well-defined monophyletic group (Erhardt 1997, Mattern 2003, Lins et al. 2017). We compare these two species with a non-crinochete oniscidean, *Ligidiumlapetum* Mulaik & Mulaik, 1942 (Ligiidae), belonging to the section Ligiamorpha and incapable of WVA (Wright, personal observation). *Armadillidiumvulgare* and *P.dilatatus* are common species in Southern California, originally introduced from Europe (Miller 1936, Arcangeli 1932, Garthwaite and Lawson 1992). *Ligidiumlapetum* is a native species in Southern California. Although scantly recorded in the literature since its original description from Central California (Mulaik and Mulaik 1942), *L.lapetum* is common in riparian oak woodlands on the south slope of the San Gabriel Mountains.

## Methods

*Armadillidiumvulgare* and *Porcelliodilatatus* were collected from the Pomona College campus and vicinity, Claremont, CA, and *Ligidiumlapetum* was collected from local foothill canyons in the San Gabriel Mountains. Animals were maintained in the lab at 22 °C in covered glass bowls with oak litter and shell fragments as a calcium source. Carrot and potato were provided *ad libitum* as supplementary food.

Isopods were examined daily for signs of molting. Pre-molting animals were identified by the appearance of the sternal calcium deposits and separated into individual 20 mL glass vials. The top of each vial was covered with 1-mm fiberglass screen mesh. Animals were maintained in controlled humidity (100 % or 97 %) by standing the inverted vials on a 4-mm steel grid of a nested sieve set (Wards, Rochester, NY). This 4-mm sieve was inserted into the bottom pan which, in turn, was filled to within 1 cm of the overlying grid with water or with saturated aqueous K_2_SO_4_ to establish a relative humidity of 100 % or 97 % respectively (Winston and Bates 1960). The top of the upper sieve chamber was then covered with a sheet of Plexiglas ringed with silicone vacuum grease. This experimental design ensured that any animal sitting on the screen mesh of a vial was no more than 1 cm from the liquid surface. Controls using a digital hygrometer probe (VWR International, San Dimas, CA) inserted through a substitute, drilled, Plexiglas cover and sealed with Parafilm showed that the relative humidity immediately above the metal grid came to within 1% of the equilibrium humidity after 15 minutes (N = 9).

Each batch of animals was weighed daily at the same time using an Ohaus digital microbalance with a resolution of 10 μg. Any fecal pellets produced were weighed separately and then discarded. Total fecal pellet mass-losses in any given 24-h period were usually less than 1 mg and few pellets were produced after animals had been isolated for 3 days. The molt stage of each animal was recorded as follows:

Premolt – sternal calcium deposits visible; recorded as days prior to the posterior ecdysis

Posterior ecdysis (PE) – posterior cuticle shed, resulting in a distinct 2-tone appearance

Anterior ecdysis (AE) – anterior cuticle shed; sternal deposits no longer visible

Postmolt – recorded as days following anterior ecdysis

The number of days pre-molt for each weighing was determined *post-facto* according to the timing of the posterior ecdysis. Following PE and AE, most animals consumed the sloughed exuvium within 2 days. Fragments of uneaten exuvia were left in the chamber and not included in mass measurements.

Since preliminary observations indicated the presence of WVA, we conducted separate trials to examine the impact of WVA on hemolymph osmolality in molting *A.vulgare*. Females undergoing parturial molts were excluded. Procedures were identical to those described above, except that animals were sampled daily for blood by puncturing the thin cuticle at the base of the 7^th^ pereopod using a pulled glass micropipette. By holding the tip in place for a few seconds prior to withdrawal, bleeding from the sample location was minimized or (in most cases) eliminated. Each sample (<20 nl) was expelled into mineral oil held in the silver sample plate of a Otago nanoliter osmometer (Otago Instruments, Dunedin, NZ), and the osmolality determined from the freezing point depression (∆T_f_):

Osmolality (Osm. kg^-1^) = ∆T_f_ / K_f_

where K_f_ is the colligative freezing point depression constant (-1.858 °C Osmol^-1^)

Although the impact of blood sampling on animal masses was small, the mass data from these animals were used solely to calculate predicted changes in osmolality (see below) and not combined with the independently collected mass-change data.

## Results

**Figure 1.**
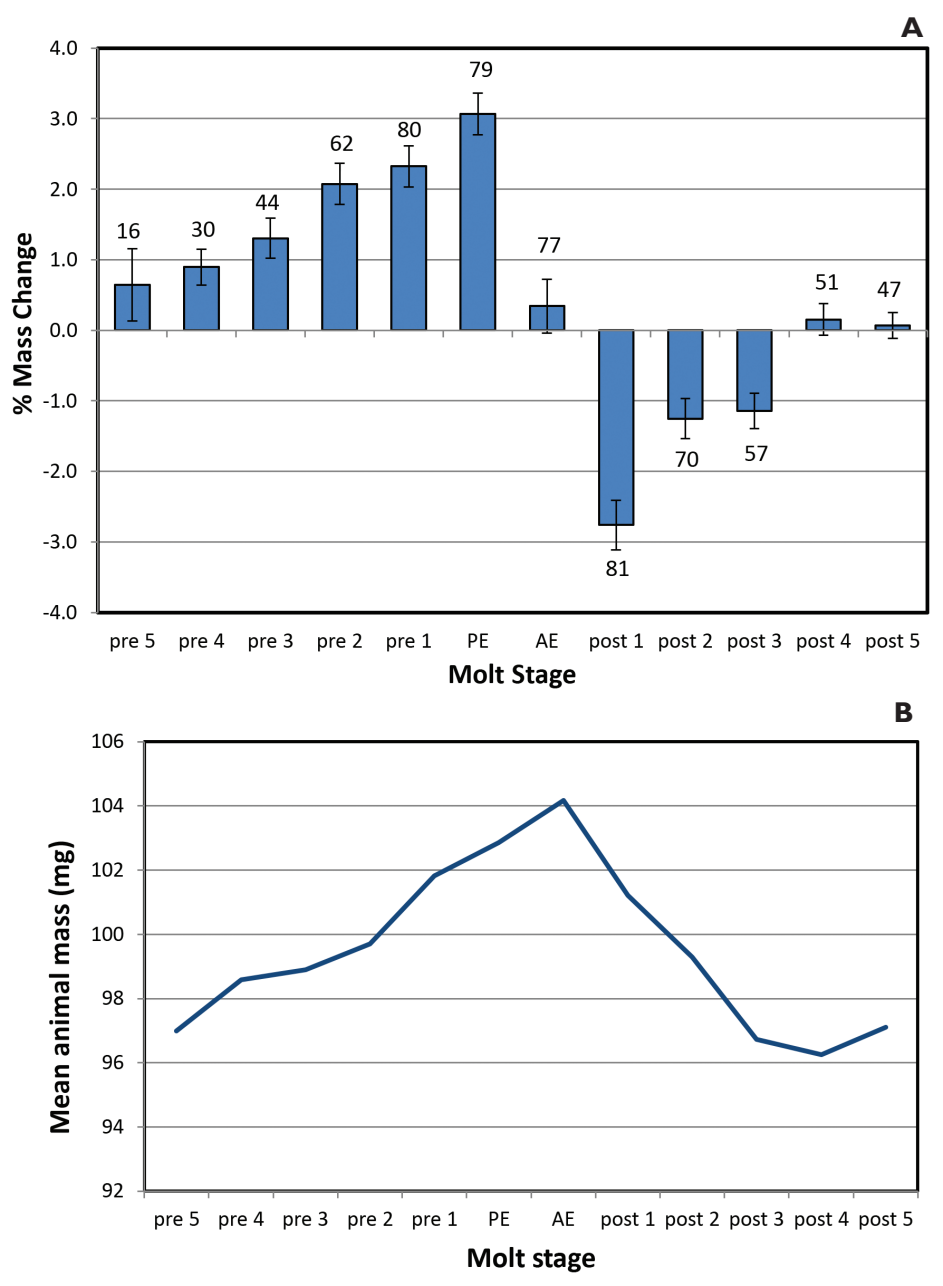
**A** Mass changes of *Armadillidiumvulgare* during molting at 100 % RH, without access to food. Pre- and post- labels refer to the number of days before/after ecdysis with data showing the % mass change over the prior 24-h period. PE = posterior ecdysis; AE = anterior ecdysis. Bars show ± SEM with sample sizes **B** Mean masses of 4 of these animals, showing the characteristic pattern of mass gain, peaking between PE and AE, followed by loss over the 3 to 4-day post-molt period.

**Figure 2.**
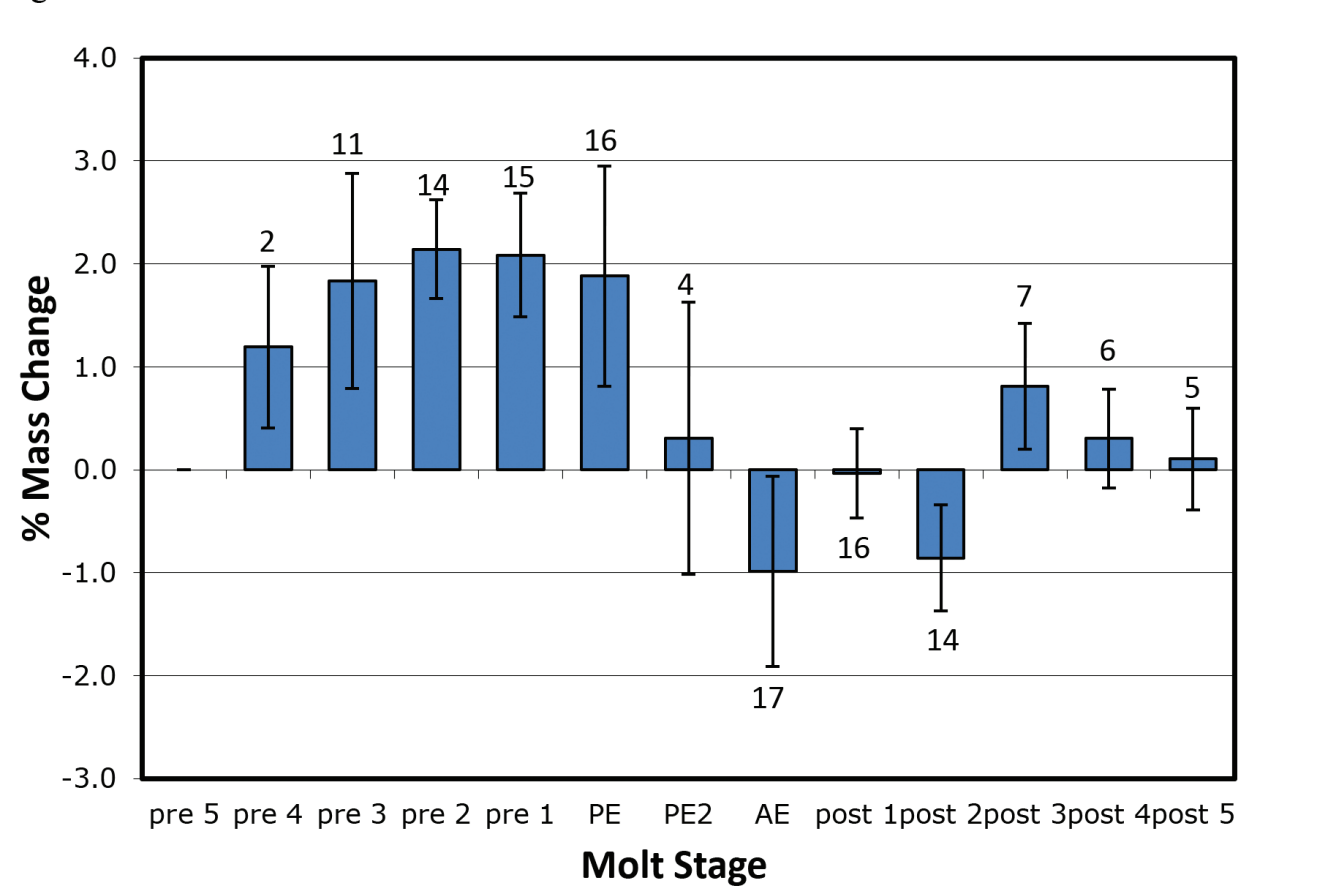
Mass changes of *Armadillidiumvulgare* during molting in 97 % RH, without access to food. Details as in Figure 1. PE2 refers to the small number of animals reaching a second day after PE without completing the anterior ecdysis.

**Figure 3.**
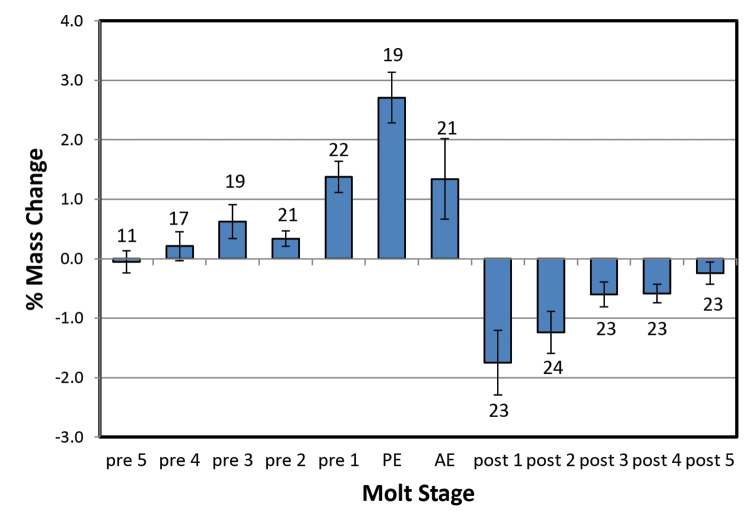
Mass changes of *Porcelliodilatatus* during molting at 100 % RH, without access to food. Bars show ± SEM with sample sizes.

**Figure 4.**
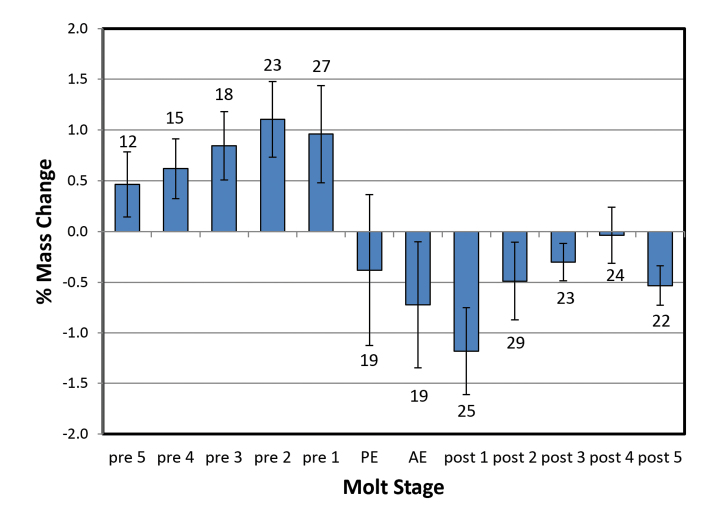
Mass changes of *Porcelliodilatatus* during molting at 97 % RH and without food. Data labels and other details as for Fig. 1.

Mass changes by day (%) for *Armadillidiumvulgare* and *Porcelliodilatatus* in 100 % and 97 % RH are shown in Figures 1–4. Several animals initiated the posterior ecdysis (PE) within 6 days of the first weighing, and a few animals died during experimentation, so sample sizes for each day are variable. In 100 % RH, both species showed a similar pattern of progressive mass increase from 5–6 days premolt, reaching a maximum of approximately 3 % d^-1^ on the day of PE. In 97 % RH, the pattern was similar but the mass increases smaller, reaching maxima of approximately 2 % d^-1^ in *A.vulgare* and 1 % d^-1^ in *P.dilatatus*, and peaking 1–2 days prior to PE. Mass gain declined sharply or reversed 1–2 days after PE and was followed by a period of water loss. This was quite variable among animals in 97 % RH, but more clearly defined in 100 % RH with the largest loss fluxes occurring in the day following anterior ecdysis (AE) and declining progressively thereafter. In a few trials with *A.vulgare*, AE occurred more than 24 h after PE; masses recorded on the second day following PE but prior to AE are denoted as PE2.

**Figure 5.**
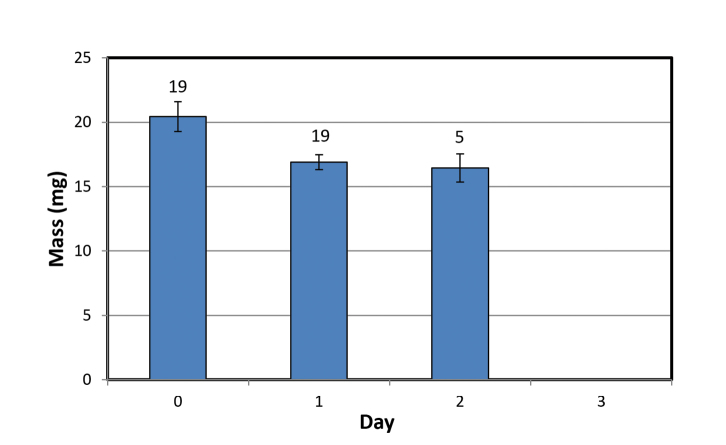
Mean masses of *Ligidiumlapetum* in 100% RH. The mean mass of the surviving animals on Day 2 is the mean % mass loss of those animals, subtracted from the mean of all animals at Day 0. No animal survived to Day 3 or initiated ecdysis.

Estimates of net mass changes over the molt period were derived by summing the daily mass changes and are presented in Table 1. Both species showed a net mass gain over the 12 to 13-day molt period. Although daily uptake fluxes were markedly smaller in 97 % RH for both species, the post-molt losses were also smaller and *A.vulgare* actually showed a larger cumulative mass gain in 97 % than in 100 % RH.

In contrast to the crinochete species, *Ligidiumlapetum* failed to show any mass gain in either 100 % or 97 % RH and no animals initiated molting. No specimen survived to Day 3 in 100 % RH (n = 12) and all animals died within 24 h in 97 % RH (n = 11). The mean mass losses after Day 1 were 15.4 % in 100% RH and 43.1 % in 97 % RH. Possible explanations for the significant mass losses in 100 %, despite the rapid equilibration time of the chamber, are considered in the Discussion.

**Figure 6.**
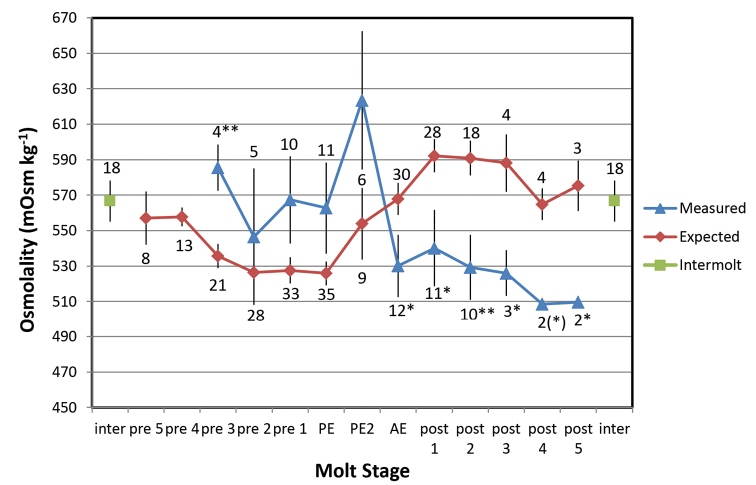
Mean measured (blue) and expected (red) values for hemolymph osmolality in *Armadillidiumvulgare* during molting in 100 % RH. Expected values are derived from the product of the mean intermolt osmolality (green symbols) and the proportional changes in blood volume over the molt cycle (see text). Bars show ± 1 SEM with sample sizes. Molt stages as in Figs 1–4. Asterisks denote significant differences between measured and expected means (2-sample t-test). * P < 0.05; ** P < 0.01. (*) P = 0.056.

Hemolymph osmolality in *A.vulgare* underwent a pronounced decline following PE2 (Fig. 6) and remained below intermolt values for the 5 days post-molt. Observed osmolalities were compared with predicted values assuming the mean osmolality as a baseline, a hemolymph mass (and volume) of 33.4 % of the hydrated intermolt animal mass (Koh and Wright 2011), and assuming that all mass changes during the molt period represent water moving into/out of the hemolymph:

**Table 1.** Cumulative mass changes (%) over the molt cycle derived from the data plotted in Figs 1–4. Data show means changes during the premolt period (6 days premolt to PE), the intramolt period (PE to AE), and the postmolt period (AE to 5 days postmolt), and the net mass gain over the total 12- to 13-day molt period. Asterisks denote a significant mass change during the respective interval (* p < 0.05; ** p < 0.005; *** p < 0.0005; 2-sample t-test).

	6-days premolt to PE	PE to AE	AE to 5-days postmolt	Net mass gain
* A. vulgare *				
100% RH	10.31***	2.72***	-4.93***	5.72
97% RH	9.13**	2.87*	0.33	8.77
* P. dilatatus *				
100% RH	5.20***	1.37*	-4.41***	2.13
97%	3.61*	0.34	2.53*	0.36

Osm._p_ = Osm._i_ . 33.4 / (∆*M*_x_ + 33.4)

Where Osm._p_ = predicted osmolality (mOsm.kg^-1^), Osm._i_ = measured intermolt osmolality (mOsm.kg^-1^), ∆*M*_x_ = proportional change in animal mass relative to intermolt mass (%), and 33.4 is the proportional volume of the hemolymph (%). The predicted variation contrasts sharply with the measured values, showing in particular markedly higher values (by 50–60 mOsm.kg^-1^) over the 6-day period following PE2. Measured osmolality is significantly elevated above predicted values at 3 days prior to PE, and significantly depressed below predicted values from AE throughout the post-molt period. The mean osmolality measured from 3–5 days post-molt is 517 mOsm.kg^-1^, representing a decrease of 8.6 % from the mean intermolt value of 567 mOsm.kg^-1^.

**Figure 7.**
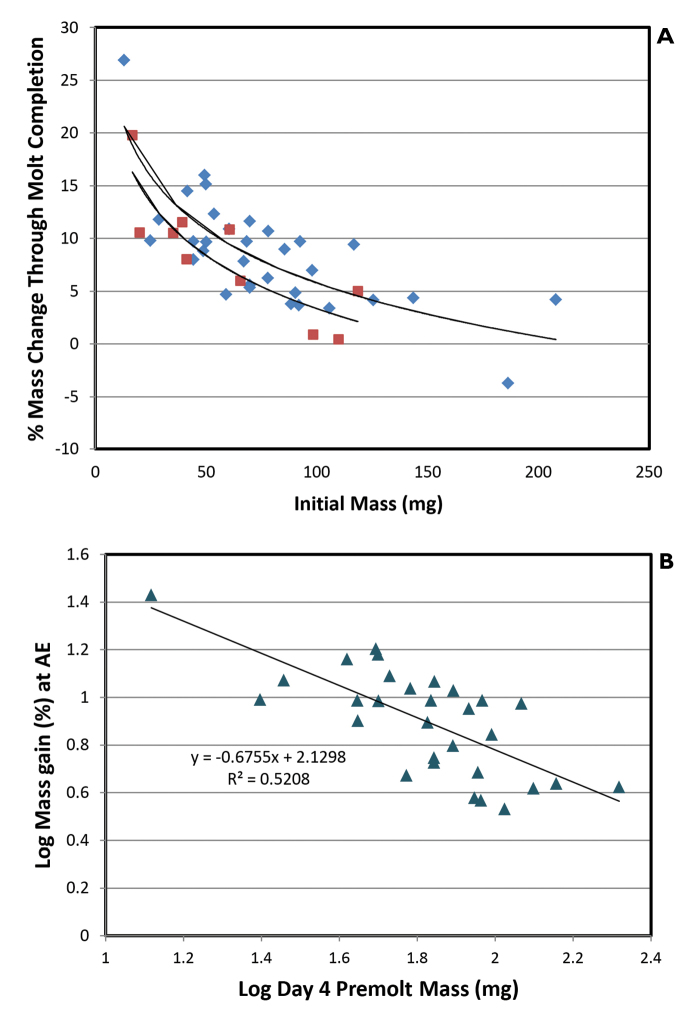
**A** Percentage mass change between 5 days premolt and anterior ecdysis for *Armadillidiumvulgare* maintained in 100 % RH (blue) and 97 % RH (red) and plotted as a function of premolt mass. Trendlines show best-fit logarithmic curves. Animals in 97 % RH achieve slightly smaller proportional mass changes to those in saturated air, consistent with the reduced vapor pressure gradient for WVA**B** Log-log plot showing the relationship between fractional mass-gain and pre-molt mass in 100 % RH (% mass gain α M^-0.676^).

Fractional mass changes of *A.vulgare* through the molting period showed an inverse logarithmic relationship to pre-molt animal mass in both 100 % and 97 % RH (Fig. 7a,b). The scaling exponent for the 100 % RH data is -0.676, indicating an area-limited process. This is considered further in the Discussion.

## Discussion

This study shows for the first time that crinochete oniscideans utilize active water vapor absorption (WVA) to increase body mass prior to ecdysis. In both 100 % and 97 % RH, *A.vulgare* and *P.dilatatus* showed a progressive increase in mass from 5 days prior to ecdysis, typically peaking on the day of ecdysis, and followed by a variable period of mass loss commencing between the posterior and anterior ecdyses. The water uptake in the first few days of weighing will actually be slightly larger than calculated here because most animals lost a small amount of mass (0.3–2.2 mg, or ca. 1–1.5 %) over this period (usually Pre5 to Pre2) in the form of fecal pellets.

The fact that water uptake is seen in 97 % RH (a_w_ = 0.97), a humidity below the equilibrium water activity of the hemolymph in *A.vulgare* and *P.dilatatus* (ca. 0.990; Wright et al. 1997), confirms the role of active WVA. Both species attained a mean water uptake of about 3 % d^-1^ in the day of PE, about 3.5 times larger than the estimated passive flux, and larger gains (up to 8.7 % d^-1^) were seen in some animals with masses in the 60–100 mg range. The increase in mass and volume due to WVA likely serves to supplement muscle contraction in generating the pulses of hydrostatic pressure that precede the pre-ecdysial transverse split in the old cuticle and the subsequent posterior and anterior ecdyses (Alikhan 1983, 1984). Critically, WVA provides a mechanism for expansion of the new cuticle and net volume increase during the molt cycle (Table 1), allowing for succeeding tissue growth during intermolt.

Notwithstanding the considerable variation among animals in maximum WVA rates, calculated values are considerably smaller than the fluxes reported by Wright and Machin (1993) in flowing air. WVA in crinochete oniscideans is potentially quite rapid, despite the high absorption thresholds, and in an RH >95 % animals can replenish significant water losses (>10 %) in a few hours. The standardized uptake fluxes for *A.vulgare* (6.7 μg h^-1^ Pa^-1^) and *P.dilatatus* (7.6 μg h^-1^ Pa^-1^), would enable proportional mass-gains of approximately 50 % in 24 h (Wright and Machin 1993). The smaller uptake rates documented here may simply reflect intermittent WVA and the need for controlled rates of cuticle expansion during the premolt. However, it is also likely that uptake fluxes are limited by the lack of air flow and regional lowering of the water vapor pressure adjacent to the condensing pleopodal surface. In that event, we would expect mass-gains under natural conditions often to occur more rapidly, and perhaps take place over fewer days, since animals frequently rest with the pleopods immediately above a saturated surface such as damp wood.

The cessation of WVA following the intramolt (between PE and AE) may be due to an inability to absorb water vapor during the period of new cuticle formation, as apparent also in *Tenebrio* larvae (Coutchié and Crowe 1979), the polyphagid cockroach *Arenivaga* sp. (Edney 1966), and the lepismatid *Thermobiadomestica* (Noble-Nesbitt 1978, 2010). In each of these insects, the capacity for WVA ceases prior to ecdysis, then resumes shortly after. In *Thermobia*, WVA resumes about 3h following ecdysis (Noble-Nesbitt 1978) while in *Tenebrio* and *Arenivaga*, the resumption takes about 2 days after ecdysis (Coutchie and Crowe 1979, Edney 1966). It is not known whether the interruption of absorption capacity is due to a need for the renewal of critical structural components of the new cuticle following apolysis or to a temporary interruption of epithelial transport processes. It is interesting, however, that WVA in *A.vulgare* and *P.dilatatus* ceases only following ecdysis, and this may be facultative rather than reflecting a loss of absorption capacity. The clear period of water loss following ecdysis is seen in both 97 % and 100 % RH and presumably serves to provide a controlled reduction of the elevated hemolymph volume and pressure generated during the premolt period. Immediately following PE, the soft posterior cuticle is expanded by the elevated hemolymph pressure and the distension of the posterior half is often striking (see also Hornung 2011). After this time, contraction of somatic muscles in the posterior segments sustains the elevated hemolymph pressure pulses and assists in the anterior ecdysis and expansion of the new anterior cuticle (Heeley 1941, Alikhan 1983, 1984), even as the animal’s net volume shows a modest decline. Calcification of the new posterior and anterior cuticle takes place rapidly, mostly occurring within 24 hours of the respective ecdyses (Steel 1993). Whether intermolt animals show significant mass and volume increase, enabled by extension of pleural and intersegmental cuticle, appears not to have been investigated.

The mass-gain prior to PE and mass-loss following AE seen in both *A.vulgare* and *P.dilatatus* differ from the post-molt volume increase described for the supra-littoral ligiid *Ligiapallasii* by Ziegler and Pennings (2000) based on hemolymph volume measurements. During the initial premolt period, hemolymph osmolality in *A.vulgare* remains elevated and higher than predicted based on the mass increase from WVA (Fig. 6), indicating that WVA is initially accompanied by compensatory mobilization of sequestered electrolytes into the hemolymph (Wright et al. 1997, Koh and Wright 2011). Hemolymph osmolality shows a sharp decrease following PE, and concentrations of specific electrolytes (Na^+^, K^+^, Mg^2+^, Cl-) have been shown to decrease in a similar pattern in *Porcellioscaber* (Ziegler and Scholz 1997). This coincides, curiously, with the onset of the mass and volume decrease and indicates a reversal of the earlier ion mobilization and re-sequestering of electrolytes following PE. The sharp divergence between observed and predicted osmolality following AE indicates that the post-molt water losses are due not to evaporation (which would be precluded in 100 % RH anyway) but to the expulsion of iso-osmotic fluid, possibly maxillary urine. This would have no net impact on hemolymph osmolality which would thus be predicted to remain unchanged following PE when vapor absorption ceases. Indeed, the expected osmolality at PE closely approximates the measured values thereafter (Fig. 6). The post-molt water losses in *A.vulgare* amount to approximately 5 % of total mass (Fig. 1, Table 1), equivalent to 15 % of the hemolymph volume. The initial water gain from WVA followed by removal of isosmotic fluid explains the net loss of solutes and decrease in hemolymph osmolality over the molt cycle; these solutes will need to be replaced during the succeeding intermolt period.

*Ligidiumlapetum* presents a clear contrast to the crinochete species, with pre-molt animals unable to molt successfully when isolated in 97 % or 100 % RH and suffering significant mass-losses even in the 100 % RH chamber. This is consistent with the absence of any WVA capacity in this species. Although some modest water loss is inevitable during chamber equilibration, an outward vapor pressure gradient would persist only for about 15 minutes in 100 % RH chamber and explains the lack of significant mass-loss between Day 1 and Day 2. It is unclear why animals did not survive beyond Day 2 in 100 % RH; this could result from prolonged effects of the initial dehydration, or additional ensuing dehydration from obligatory intermittent production of maxillary urine (Hoese 1981).

The question remains as to how *Ligidium* spp. and other non-crinochete terrestrial oniscideans achieve volume expansion to enable molting in the absence of WVA. We have found *L.lapetum* only in close proximity to liquid water, inhabiting litter and humic soil in the riparian fringe. Here it has ready access to freshwater and could drink or possibly take up water via the uropods and rectum, as documented for the Crinocheta (Spencer and Edney 1955, Drobne and Fajgelj 1993), to bring about pre-molt expansion. The Ligiidae also depend on an external freshwater source to provision the marsupial fluid (Hoese 1981, Yoshizawa and Wright 2011). The other major radiation of terrestrial oniscideans is the family Trichoniscidae, mostly small animals with little resistance to water loss. Trichoniscids are primarily endogean in habit, inhabiting damp soil, decomposing wood and litter (see Sutton 1980, and references therein). Standen (1970) showed that *Trichoniscuspusillus* was unable to gain mass during the molt in the absence of liquid water. Trichoniscids may imbibe soil capillary water as a means of volume expansion during the molt. However, their high permeability and small size would also allow them to gain water quite rapidly through passive diffusion in saturated conditions. Proportional water fluxes measured for 3 species range from 44 to 59 % h^-1^ a_w_^-1^ (Wright and Machin 1993) which translate to approximately 10–15 % mass gain per day in saturated air. Trichoniscids may be able to regulate mass gain and loss during the molt cycle by exploiting passive diffusional gain if essential water losses from maxillary urine can be limited accordingly.

*A.vulgare* females attain reproductive maturity within the first year and may live for up to 4 years (Lawlor 1976). Animal masses in the current study varied appreciably and will account for some of the variance among the mass exchange data. Mass-change of oniscideans during growth is approximately linear as a function of time (Hubbell 1971, Helden and Hassall 1998), so proportional mass gain (∆*M/M*, %) will vary over time as the function *M*^-1^. This function is the product of the mass-gain over one molt cycle and the intermolt period. To explore this relationship further, we analyzed the proportional mass gain (∆*M/M*) at anterior ecdysis as a function of the preceding (intermolt) animal mass (*M*, mg) for *A.vulgare*. We used the mass gain at AE rather than the net mass gain over the complete molt cycle owing to the much larger sample size. Results are shown in Figure 7 (a, b). In both 100 % and 97 % RH, ∆*M/M* decreases exponentially as a function of mass, and log transformation (Fig. 7b) yields the following relationships:

100 % RH ∆*M/M* = 135.*M*^-0.676^ (n = 30; r^2^ = 0.52)

97 % RH ∆*M/M* = 78.*M*^-0.564^ (n = 8; r^2^ = 0.71)

Although the sample size for 97 % RH is small, the reasonable congruence of the two exponents supports the assertion that the relative mass gain prior to molt scales with an exponent of -0.67, and mass-gain (∆*M*) scales as *M*^0.33^ (or *L*^1^ where L is length). This indicates that oniscideans follow the Brooks-Dyar Law (Dyar 1890, Daly 1985), showing a regular, geometric increase in the linear dimensions of sclerotized parts of the integument at each molt (∆*L*). It further shows that intermolt period must scale as M^0.67^ (∆*L*^2^) in order for mass to increase linearly.

To our knowledge, these crinochete isopods represent the first demonstrated instance of WVA functioning in volume increase during molting. Given the limited means of volume expansion available to terrestrial taxa, however, this may be a widespread function of WVA. Further work examining such a role in other vapor-absorbing groups would be revealing, as would studies of volume regulation during molting in arachnids and myriapods where the mechanisms remain largely elusive.

## Conclusion

*Armadillidiumvulgare* and *P.dilatatus* show a progressive increase in mass in the absence of food or liquid water from 5–6 days prior to the posterior ecdysis. This mass-gain is seen in 100 % RH or 97 % RH, confirming the role of active water vapor absorption. Following the anterior ecdysis, both species show a variable period (3–4 days) of mass-loss accompanied by loss of ions from the hemolymph. The net mass and volume gain over the premolt period could supplement pulses of hemolymph pressure to bring about the anterior and posterior ecdyses and, critically, will allow for volume expansion and growth of normally sclerotized and/or mineralized cuticle. The ligiid, *Ligidiumlapetum*, lacks the capacity for WVA and lost mass over the molt cycle, even in 100%. This species presumably depends on liquid water uptake for volume expansion.
